# Ectomycorrhizal fungi reduce the light compensation point and promote carbon fixation of *Pinus thunbergii* seedlings to adapt to shade environments

**DOI:** 10.1007/s00572-017-0795-7

**Published:** 2017-08-24

**Authors:** Liang Shi, Jie Wang, Binhao Liu, Kazuhide Nara, Chunlan Lian, Zhenguo Shen, Yan Xia, Yahua Chen

**Affiliations:** 1 College of Life Sciences, Nanjing Agricultural University, Nanjing, 210095 China; 2 Department of Natural Environmental Studies, Graduate School of Frontier Science, The University of Tokyo, 5-1-5 Kashiwanoha, Kashiwa, Chiba 277-8563 Japan; 3 Asian Natural Environmental Science Center, The University of Tokyo, 1-1-8 Midoricho, Nishitokyo, Tokyo 188-0002 Japan

**Keywords:** Ectomycorrhiza, Photosynthetic rate, Light compensation point, Japanese black pine, Light limitation

## Abstract

We examined the effects of three ectomycorrhizal (ECM) symbionts on the growth and photosynthesis capacity of Japanese black pine (*Pinus thunbergii*) seedlings and estimated physiological and photosynthetic parameters such as the light compensation point (LCP), biomass, and phosphorus (Pi) concentration of *P. thunbergii* seedlings. Through this investigation, we documented a new role of ectomycorrhizal (ECM) fungi: enhancement of the survival and competitiveness of *P. thunbergii* seedlings under low-light condition by reducing the LCP of seedlings. At a CO_2_ concentration of 400 ppm, the LCP of seedlings with ECM inoculations was 40–70 μmol photons m^−2^ s^−1^, significantly lower than that of non-mycorrhizal (NM) seedlings (200 μmol photons m^−2^ s^−1^). In addition, photosynthetic carbon fixation (Pn) increased with light intensity and CO_2_ level, and the Pn of ECM seedlings was significantly higher than that of NM seedlings; *Pisolithus* sp. (Pt)- and *Laccaria amethystea* (La)-mycorrhizal seedlings had significantly lower Pn than *Cenococcum geophilum* (Cg)-mycorrhizal seedlings. However, La-mycorrhizal seedlings exhibited the highest fresh weight, relative water content (RWC), and the lowest LCP in the mycorrhizal group. Concomitantly, ECM seedlings showed significantly increased chlorophyll content of needles and higher Pi concentrations compared to NM seedlings. Overall, ECM symbionts promoted growth and photosynthesis while reducing the LCP of *P. thunbergii* seedlings. These findings indicate that ECM fungi can enhance the survival and competitiveness of host seedlings under low light.

## Introduction

Mycorrhizal fungi play crucial roles in shaping the development of forest ecosystems (Clark and St Clair 2011). In forests, light is the greatest limiting factor for seedling survival and growth (Kolb et al. 1990). Today, conifer trees within native ranges often account for less than 15% of standing stock, primarily due to shade intolerance and slow initial growth rates (Kabrick et al. 2015). In old-growth forests of Eastern Asia, *Taiwania cryptomerioides* is a shade-intolerant and long-lived conifer that experiences intense light competition, yet eventually emerges from the canopy (40–70 m), which comprises more shade-tolerant evergreen broad-leaved trees (He et al. 2015). Paper birch also competes well for light and inhibits the growth of shade-intolerant conifers (Callaway and Walker 1997). These previous studies indicate that some conifer species do not possess a competitive advantage in forest ecosystems under natural conditions. Therefore, the protection and restoration of shade-intolerant conifers remain challenging but essential endeavors.

*Pinus thunbergii* Parlat. is a major shade-intolerant evergreen conifer species in Japan and has been introduced to China and America (Choi 1986; Masaka et al. 2010). Taniguchi et al. (2008) reported that when *P. thunbergii* seedlings were planted in soil obtained from a black locust-dominated (*Robinia pseudoacacia*) area, all seedlings died under low-light intensity conditions. Furthermore, inhibition of the regeneration of *P. thunbergii* seedlings was strongly mediated by shading (Taniguchi et al. 2007). To adequately protect and restore this species, it is critical to improve the ability of *P. thunbergii* seedlings to survive under light-limiting conditions by enhancing their shade tolerance. A key trait affecting the survival of seedlings grown under light-limiting conditions is the light compensation point (LCP) (Kitao et al. 2016), i.e., the light intensity at which the photosynthetic rate of plant leaves is equivalent to the respiration rate (Taiz and Zeiger 2010). Although the benefits of mycorrhizal symbionts are well established, whether ectomycorrhizal (ECM) fungal inoculation improves the capacity of *P. thunbergii* seedlings to utilize low light has not been examined.

Photosynthesis is the process by which plants convert carbon dioxide (CO_2_) and water into sugars and oxygen using solar energy; this reaction is highly sensitive to environmental changes (Taiz and Zeiger 2010). In some broad-leaved plants, such as poplars and Mediterranean orchids, mycorrhizal fungi effectively improve Pn and leaf chlorophylls *a* and *b*, as well as the maximal photochemical efficiency (F_v_/F_m_) of host plants (Gambini and Vellini 2007; Smith and Read 2008). Similarly, the ECM plants of *Helianthemum sessiliflorum* exhibited higher rates of photosynthesis (35%), transpiration (18%), and dark respiration (49%) than non-mycorrhizal plants (Turgeman et al. 2011). Most previous studies have focused on the effects of ECM inoculation on the salt tolerance of *P. thunbergii* seedlings (Kim et al. 2016) or their resistance to damage caused by pathogenic microorganisms (Ichihara et al. 2001) because of its high salt tolerance (Kim et al. 2016) and high capacity to intercept salt spray (Kim 2010), and a few studies have examined the photosynthetic responses of ECM *P. thunbergii* (Nazir and Khan 2012; Kubota et al. 2017). However, the limited study to date has investigated the shade tolerance of ECM *P. thunbergii* seedlings. Meanwhile, the response of soil to increased carbon availability is largely driven by root-associated ECM fungi in forest ecosystems, as they partition host-derived carbon belowground (Fransson 2012). Aspen trees with mycorrhizal associations exhibit higher net photosynthetic rates and an ability to maintain higher sucrose levels in their leaf tissue compared to those without mycorrhizal associations (Einig et al. 1997; Loewe et al. 2000). Furthermore, shading can lead to carbon limitation in aspen through decreased photosynthetic rates (Calder et al. 2011). For example, light reductions and/or shifts in soil chemistry limited height growth, biomass gain, photosynthesis, and the production of defense compounds (phenolic glycosides and condensed tannins).

Photosynthetic parameters such as CO_2_ assimilation (A), actual PS-II efficiency (Φ_PS II_), gas exchange, and needle necrosis have typically been the foci of previous studies (Bucking and Heyser 2003; Nguyen et al. 2006; Heinonsalo et al. 2015). Among these parameters, determining in vitro Pn in conifers often involves cutting branches (Zeibig et al. 2005; Renninger et al. 2013), isolating needle chloroplasts (Huang and Tao 2004), or collecting needles from seedlings in a leaf chamber cuvette (Thompson and Wheeler 1992; Ibell et al. 2013). However, these photosynthetic parameters, including in vitro LCP, may not be accurate. Some studies have shown that leaves collected in vitro make their water supply be interrupted, leading to the limitation of stomatal or non-stomatal factors (Saliendra et al. 1995; Flexas and Medrano 2002), stomatal closure, reducing adenosine triphosphate (ATP), and ribulose-1,5-diphosphate (RuBP) levels, decreasing content and activity of ribulose-1,5-biphosphate carboxylase/oxygenase (Rubisco) and reducing the photosynthetic rate ultimately (Gimenez et al. 1992; Flexas and Medrano 2002).

In this study, our objectives were to determine (1) whether inoculation with ECM fungi improves the capacity of *P. thunbergii* seedlings to utilize low light; (2) how ECM fungal inoculation enhances the shade tolerance of *P. thunbergii* seedlings; and (3) the effects of ECM fungi on the carbon fixation (Pn) of *P. thunbergii* seedlings.

## Materials and methods

### Preparation of ectomycorrhizal *P. thunbergii* seedlings

#### Source of strain

Three isolates of ECM fungi, *Cenococcum geophilum* (KY075873), *Pisolithus* sp. (KY075875), and *Laccaria amethystea* (KY075878), were obtained from Sanqing Mountain in Jiangxi Province, China (Table 1). These fungi were chosen because they are easily cultivated, grow rapidly, and readily form ECM associations. Furthermore, they are globally distributed, and they maintain associations with a broad range of tree species, including *P. thunbergii*.

**Table 1:** Sources of and information regarding ECM fungal strains used in this study

Strains	Sites	Isolation source	Lat and Lon	Sequence ID
*Cenococcum geophilum*	Mountain Sanqing	Mycorrhizal root tips	29.03 N 118.26 E	KY075873
*Pisolithus* sp.	Mountain Sanqing	Sporocarps	28.54 N 118.03 E	KY075875
*Laccaria amethystina*	Mountain Sanqing	Sporocarps	28.55 N 118.09 E	KY075878

#### Preparation of mother seedlings

After germination for 40 days, non-mycorrhizal (NM) *P. thunbergii* seedlings were transplanted into PVC tubes filled with an autoclaved (121 °C, 3 h) mixture (1:1, *v*/*v*) of Tanashi nursery soil (black sand loam) and Shibanome soil (volcanic sand; Setogahara Co., Gunma, Japan) with half the volume of the tube. The physicochemical properties of these soils have been published elsewhere (Chen et al. 2015). For the inoculation treatments, Pt-, Cg-, or La-agar fungal inoculum plugs (1 cm diameter) were cut from the mycelial edge of actively growing fungi and then put between roots of *P. thunbergii* seedlings. Seedlings were grown in a temperature-regulated greenhouse at 25 °C during the day and 20 °C at night for 4 months. ECM colonization of the root tips of every seedling was examined by stereomicroscopy. Based on the yellow (Pt), black (Cg), and purple (La) mantle and mycelial color, neither cross-contamination nor contamination by other fungi was observed. Three ECM treatments were established as the Pt-, Cg-, and La-mother seedlings, and all treatments were repeated three times. Mother seedlings with well-developed ECM roots (nearly 80% colonization on fine roots) and abundant external mycelia in soil were used for preparation of offspring seedlings. Non-mycorrhizal mother seedlings were cultivated under the same growth conditions as mycorrhizal mother seedlings. No ECM root tips were observed on the latter seedlings by stereomicroscopy.

#### Preparation of offspring seedlings

**Fig. 1:**
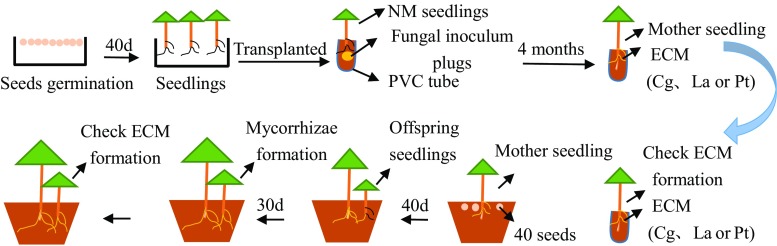
Schematic diagram of the preparation process of mother and offspring seedlings

Three individuals of each type of mother seedling (Pt-, Cg-, La-mycorrhizal) or NM were transplanted into evenly spaced individual plastic pots (20 cm diameter, 16 cm depth), which were filled with an autoclaved (121 °C, 3 h) mixture (1:1, *v*/*v*) of Tanashi nursery soil (black sand loam) and Shibanome soil (volcanic sand; Setogahara Co., Gunma, Japan). Nutritional status of soils included 2.36 ± 0.11 g kg^−1^ total N, 73.0 ± 5.6 mg kg^−1^ available N, 876 ± 44 mg kg^−1^ total P, and 2.1 ± 0.1 mg kg^−1^ available P. *P. thunbergii* seeds were surface sterilized with 30% H_2_O_2_ for 15 min, rinsed with deionized water, and then uniformly sowed around the mother seedlings in each pot (40 seeds/pot). After 40 days, these offspring seedlings were thinned to 30 per pot, maintaining an approximately uniform size. Seedlings were cultivated in an incubator (65 ± 10% relative air humidity, 1500 μmol photons m^−2^ s^−1^, 25 °C/20 °C, with a 16-h light/8-h dark photoperiod) for another 30 days to allow mycorrhizal formation. After checking ECM formation and ECM colonization rates, offspring seedlings of ECM group with well-developed ECM roots (nearly 80% colonization) and offspring seedlings of NM-group with no ECM roots were used to analyze Pn, Pi concentration, plant biomass, relative water content (RWC), and chlorophyll content in needles. All treatments were repeated three times in three independent experiments. The preparation process of mother and offspring seedlings is illustrated in Fig. 1.

### Photosynthetic rate measurement

Non-destructive in situ measurements of Pn were performed using a portable photosynthesis system (LI-6400, Li-COR, Lincoln, NE, USA) equipped with a conifer chamber (Li-COR, LI-6400-05). Whole needles of individual seedlings were placed inside the conifer chamber. To determine the LCP and light saturation point (LSP), we derived light response curves by inserting translucent cardboard between the light source and the seedlings to vary photosynthetic active radiation at 0, 20, 40, 70, 120, 150, 170, 190, 220, 250, 300, 400, 500, 600, 800, 1000, 1200, 1500, 1800, 2200, and 2600 μmol photons m^−2^ s^−1^ while maintaining CO_2_ concentrations at 400 ppm. Resulting data were used to fit the photosynthetic curve. The intersection of the curve and the *x*-axis corresponds to the LCP. To measure the CO_2_ saturation point (CSP), the CO_2_ concentration was adjusted to 0, 50, 100, 150, 200, 300, 400, 600, 800, 1000, and 1200 ppm using CO_2_ gas cylinders while light intensity was maintained at 1000 μmol photons m^−2^ s^−1^. These measurements were taken within a LI-6400 portable photosynthetic measurement apparatus equipped with a conifer chamber (Li-COR, Lincoln, NE, USA). The temperature inside the chamber was maintained at 25 °C, and measurements were taken after each change in CO_2_ to allow the adjusted conditions to stabilize in the chamber. Assimilation rates are expressed as per dry needle weight, and data are presented as averages and standard division. The pot experiments were set using a two-way crossed factorial design: (1) 21 levels of absorbed light treatments × four ECM treatments (three inoculation treatment groups and a control group) and (2) 11 levels of ambient CO_2_ concentration × four ECM treatments; each experiment had three replicate pots per treatment.

#### Determination of biomass and chlorophyll content in *P. thunbergii* seedlings

The entire root system of each seedling was removed from the soil and gently washed with tap water followed by deionized water. Fresh and dry weights were measured; determination of relative moisture content (RWC) was according to Jolly et al. (2014). To measure chlorophyll content, needles were cleaned with absorbent paper; cut needles (0.2 g) were weighed and put in a test tube with a 2:1 mixture of acetone and ethanol. Samples were kept in the dark at room temperature for 16 h for chlorophyll extraction. UV spectrophotometry was used to determine the light absorption (Hiscox and Israelstam 1979).

#### Phosphorus concentration in *P. thunbergii seedlings*

Plant materials were oven dried at 80 °C for 72 h and milled into a fine powder using a mortar and pestle. The powdered plant material was wet digested in heat-resistant glass tubes on a heating block using an 87:13(*v/v*) mixture of nitric acid and perchloric acid, and the digests were dissolved in 5% HNO_3_ to analyze Pi content. The Pi concentration in the solution was determined colorimetrically using the phosphomolybdate method (Murphy and Riley 1962). To ensure accuracy, we processed and analyzed apple leaves (Certified Standard reference materials 1515, USA) as a duplicated reference material in the same way. Reagent blanks and analytical duplicates were used when appropriate to ensure accuracy and precision in the analysis. The Pi recovery rates in the reference materials were 95–105%.

### Statistical analyses

Data were statistically analyzed using SPSS software. Two-way ANOVA was used to determine significant differences between the photosynthetic carbon fixation of seedlings and ECM inoculation. In addition, we used one-way ANOVA and Tukey’s post hoc test at *P* < 0.05 to evaluate the effects of ECM inoculation on the biomass, chlorophyll content, Pi concentration, and moisture of seedlings. Significant differences are indicated, and all values represent the mean ± SD of three replicates.

## Results

### LCP and photosynthetic rates of *P. thunbergii* seedlings

**Fig. 2:**
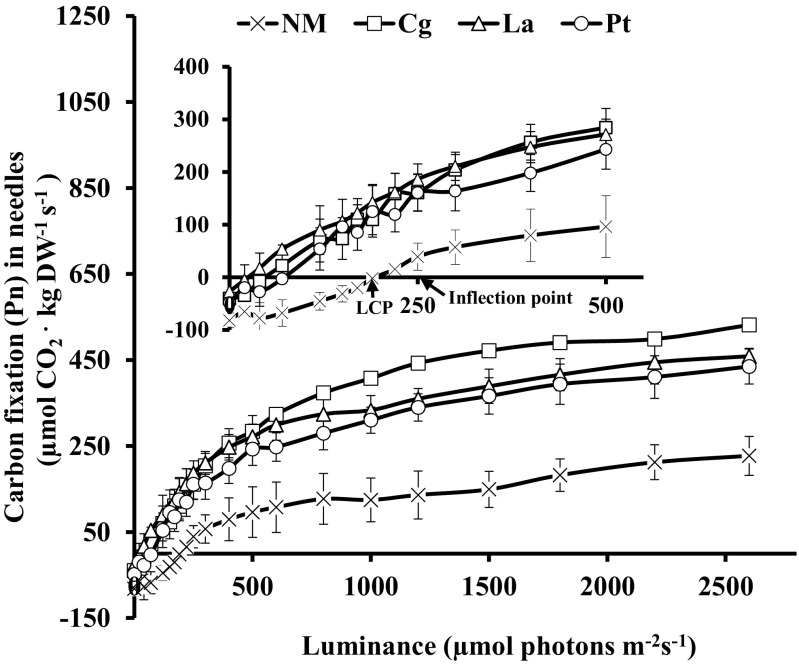
Carbon fixation rates (Pn) of *P. thunbergii* seedlings inoculated with *C. geophilum* (Cg), *Pisolithus* sp. (Pt), or *L. amethystea* (La) and in non-mycorrhizal (NM) seedlings under increasing luminance (μmol photons m^−2^ s^−1^) at 400 ppm CO_2_

The LCP significantly differed between NM and ECM *P. thunbergii* seedlings (Fig. 2). In NM plants, the LCP occurred at a luminance of about 200 μmol photons m^−2^ s^−1^, whereas the LCP of ECM seedlings ranged from 40 to 70 μmol photons m^−2^ s^−1^, depending on the fungal species (Fig. 2). In addition, luminance above the LCP resulted in a proportional increase in Pn (Fig. 2). The Pn of needles increased with luminance from 0 to 2600 μmol photons m^−2^ s^−1^, and photosynthesis saturation did not occur. However, near the inflection point, the Pn of needles slowed down. Furthermore, the Pn of ECM seedlings was significantly higher than that of NM seedlings at the same light level. For example, when luminance was 800 μmol photons m^−2^ s^−1^, the Pn of NM seedlings was 127.0 μmol CO_2_ kg (FW)^−1^ s^−1^ compared to 373.6, 324.3, and 279.8 μmol CO_2_ kg (FW)^−1^ s^−1^ for Cg, La, and Pt seedlings, respectively. Cg-mycorrhizal seedlings had the highest Pn among the three fungal species (Fig. 2).

### Photosynthetic responses to CO_2_

**Fig. 3:**
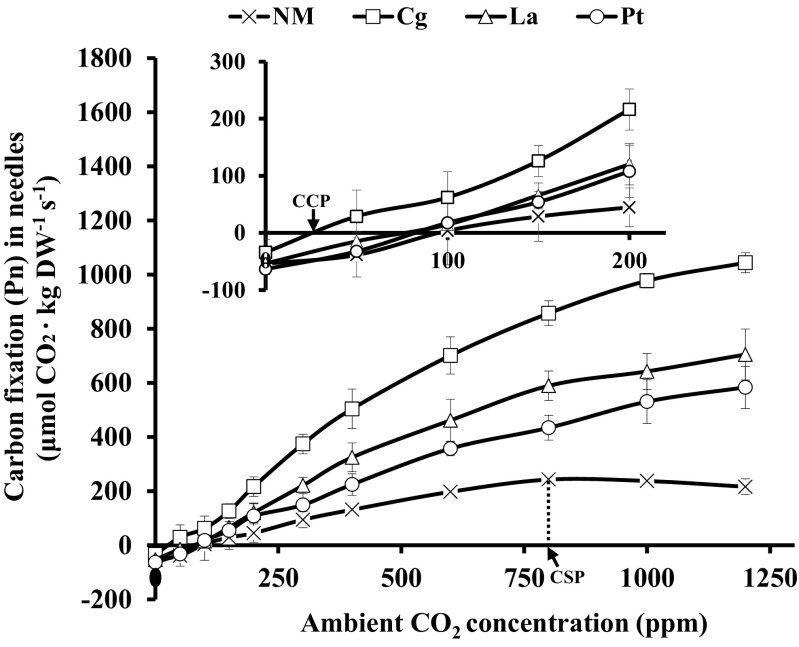
Carbon fixation rates of *P. thunbergii* seedlings inoculated with Cg, Pt, or La ECM and in non-mycorrhizal (NM) seedlings subjected to a range of ambient CO_2_ levels at a light intensity of 1000 μmol photons m^−2^ s^−1^

When photosynthetic active radiation was 1000 μmol m^−2^ s^−1^, higher CO_2_ concentrations supported higher Pn (Fig. 3). Generally, the Pn of Cg-mycorrhizal seedlings was the highest, followed by that of La- and Pt-mycorrhizal seedlings, and the Pn of NM seedlings was the lowest. When CO_2_ levels reached 836 ppm, NM seedlings experienced CO_2_ saturation, whereas the Pn of ECM seedlings did not indicate CO_2_ saturation throughout the experiment. In addition, the Pn of ECM seedlings was significantly higher than that of NM seedlings at certain CO_2_ concentrations. For example, at the current approximate atmospheric CO_2_ concentration of about 400 ppm, the Pn values of Cg-, La-, and Pt-mycorrhizal seedlings were 3.8, 2.5, and 1.7 times higher than that of NM seedlings, respectively. Moreover, when CO_2_ was 1200 ppm, the Pn values of Cg-, La-, and Pt-mycorrhizal seedlings were 4.8, 3.3, and 2.7 times higher than that of NM seedlings, respectively. These findings indicate that ECM fungi inoculation significantly improve the CO_2_ fixation (Pn) of *P. thunbergii* seedlings under different CO_2_ concentrations.

### Effects of ECM inoculation on biomass, chlorophyll content, Pi concentrations, and relative water content of *P. thunbergii* seedlings

**Fig. 4:**
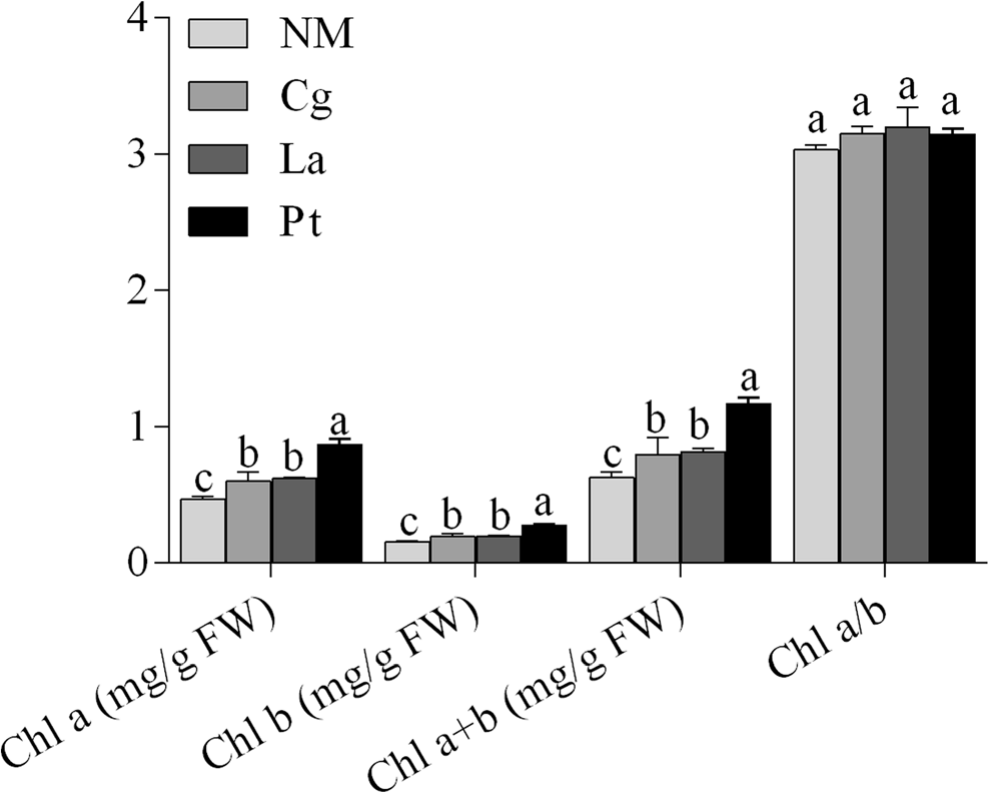
Effects of ECM inoculation on chlorophyll content and the chlorophyll *a*/chlorophyll *b* ratio in needles of *P. thunbergii* seedlings

ECM seedlings had significantly greater fresh weight, dry weight, chlorophyll content, phosphorus concentrations, and relative water content compared to NM seedlings (Table 2). The fresh weights and relative water content (RWC) of La seedlings were significantly higher than those of Cg and Pt seedlings, which corresponded to increases of 93.3 and 29% over NM, respectively; but dry weight did not significantly differ among the ECM groups, and Pt-, Cg-, and La-mycorrhizal seedlings corresponded to increases of 60.4, 54.8, and 71.7% over NM, respectively (Table 2). However, Pt-mycorrhizal seedlings had the highest chlorophyll *a*, chlorophyll *b*, and total chlorophyll content among the ECM treatments. The chlorophyll *a*/*b* ratio in needles did not significantly differ between NM and ECM seedlings (Fig. 4). Pi concentrations of Pt-, Cg-, and La-mycorrhizal seedlings respectively corresponded to increases of 79.0, 69.6, and 73.1% over NM seedlings and did not significantly differ among ECM groups (Table 2).

**Table 2:** Effects of ECM inoculation on biomasses, Pi concentrations, and relative water content of *P. thunbergii* seedlings

Plant	Plant fresh weight (mg)	Plant dry weight (mg)	Pi concentrations (mg/kg DW)	Relative water content (%)
Non-mycorrhizal (NM)	299.2 ± 20.0c	24.1 ± 1.6b	695.8 ± 58.8b	60.4 ± 1.7c
Cg-mycorrhizal (Cg)	505.5 ± 27.9b	37.3 ± 2.1a	1179.7 ± 121.2a	78.5 ± 1.8b
Pt-mycorrhizal (Pt)	493.1 ± 25.7b	38.6 ± 2.0a	1245.3 ± 55.2a	79.0 ± 0.9b
La-mycorrhizal (La)	578.2 ± 43.2a	41.3 ± 3.1a	1204.7 ± 103.2a	88.8 ± 1.3a

Different lower case letters indicate statistically significant differences (*p* < 0.05)

## Discussion

In the present study, we analyzed whether inoculation with ECM fungi improved the capacity of *P. thunbergii* seedlings to utilize low light. The results showed that ECM inoculation significantly improved the photosynthetic rates and reduced the LCP of *P. thunbergii* seedlings (Fig. 2). These results suggest that the three types of mycorrhizal *P. thunbergii* plants were able to photosynthesize at lower levels of light compared to NM plants. Most studies explored the effects of shading on the growth, C partitioning, or ECM colonization of pine seedlings (Dehlin et al. 2004; Warren et al. 2012; Trocha et al. 2016). However, studies on the effect of ECM fungi on shade tolerance and photosynthetic efficiency of pine trees remain scarce. The present work provides novel evidence that ECM fungi can significantly improve the capacity of *P. thunbergii* seedlings to maximize their use of limited light.

How does ECM fungal inoculation enhances the shade tolerance of *P. thunbergii* seedlings? Several factors may help to explain the observed changes in light utilization efficiency. For example, a nutritional effect due to improved nutrient status of ECM plants, particularly Pi absorption, is a well-known phenomenon (Smith and Read 2008), and increases in net photosynthesis following increases in Pi have been reported for various species. Wu et al. (2012) demonstrated that enrichment of CO_2_ or inorganic Pi levels in the culture medium significantly increased specific growth rate, light-saturated photosynthesis, and photosynthetic efficiency but tended to decrease dark respiratory rate, saturating irradiance for photosynthesis, and LCP values. The addition of Pi (either alone or with nitrogen) improved the photosynthetic capacity of the grass *Bothriochloa ischaemum* under 40 and 20% water regimes by increasing net photosynthetic rate, light-saturated net photosynthetic rate, and apparent quantum efficiency, while reducing dark respiration rate and LCP (Xu et al. 2013). Consistent with our first hypothesis, our results indicated that the three types of ECM fungi increased Pi levels and reduced the LCP of *P. thunbergii* seedlings, the latter being one of the most important factors enhancing photosynthesis, thus allowing *P. thunbergii* seedlings to adapt to low-light conditions. Similarly, numerous studies have also shown that light limitation leads to a decrease in plant biomass (Wilkinson et al. 2012) and reduces the Pi transfer to the host plant (Nehls et al. 2007). These observations suggest that under low-light conditions, plants allocate proportionally less carbon to the roots. In our study, the three types of ECM *P. thunbergii* seedlings exhibited higher carbon fixation efficiencies (Table 2) and lower LCP compared to NM seedlings. These results indicate that even under low-light conditions, the ECM seedlings maintained higher carbon fixation capacity and stronger shade tolerance compared to controls. ECM may confer this ability via increased host plant absorption of water and nutrients, especially Pi, thus enhancing the dry matter accumulation of *P. thunbergii* seedlings (Table 2).

On the other hand, non-nutritional factors may also influence light utilization efficiency via changes in chlorophyll content of *P. thunbergii* needles. Plants grown under shaded conditions optimize light absorption efficiency by increasing pigment density per unit leaf area (Wittmann et al. 2001). Song and Wu (2011) documented that inoculating *Populus* seedlings with *Lactarius insulsus* maximized both actual photochemical efficiency and chlorophyll *a* and *b* contents. Our results are consistent with these previous findings; moreover, chlorophyll *a* and *b* contents in Pt-mycorrhizal seedlings were significantly higher than those in Cg- or La-mycorrhizal seedlings (Fig. 4). Sun et al. (2016) reported that needle chlorophyll content responded positively to increased canopy openness. Under low-light conditions, shade-tolerant plants, such as *Athyrium pachyphlebium*, exhibit greater efficiency in absorbing and utilizing light energy via increases in chlorophyll *b* (Huang et al. 2011). Although the chlorophyll content of plant leaves may sometimes be lower than that of leaves grown under higher light, ECM fungi may improve the shade tolerance of *P. thunbergii* seedlings by fueling increases in chlorophyll content and allowing for more effective photosynthesis.

In addition, the effects of ECM fungi on the carbon fixation (Pn) of *P. thunbergii* seedlings have been studied. The regulation of photosynthesis is limited by demand for carbohydrates and provides the basis for the source–sink concept of carbon metabolism (Paul and Foyer 2001). Previous studies have shown that substantial amounts of carbon flow through mycorrhizal mycelia to different components of the soil ecosystem, and the cost of maintaining ECM associations has been estimated to range between 15 and 28% of net carbon fixation (Orwin et al. 2011; Nasholm et al. 2013). In the ECM symbiosis, up to one third of plant photoassimilates can be transferred to the fungal partner (Nehls et al. 2007). In our study, the three types of ECM fungi significantly improved carbon fixation of *P. thunbergii* seedlings (Fig. 2), which may have been driven by increased host seedling absorption of water and Pi (Bucking and Heyser 2003; Heinonsalo et al. 2015) in addition to enhanced chlorophyll content of the needles of ECM seedlings. The higher fresh weight, RWC, and the lower carbon fixation of La-mycorrhizal seedlings than Cg-mycorrhizal seedlings (Table 2) suggest that carbon transfer between ECM tree species primarily occurs through a direct hyphal pathway Simard et al. (1997). Neumann and Matzner (2014) reported that ECM plants had higher respiratory rates compared to NM plants. Together, these two mechanisms explained that ECM seedlings have more energy compared with NM seedlings, as a result of more photosynthate output and more transported carbon to roots and reduce feedback inhibition of photosynthesis. Compared to Cg-mycorrhizal seedlings, La-mycorrhizal seedlings exhibited higher respiration rates and RWC (Table 2), both of which are involved in photosynthesis.

In summary, we first determined the light and CO_2_ response curves of photosynthesis in *P. thunbergii* seedlings. Our results illustrated that the three ECM fungi studied here are likely involved in mediating several beneficial responses to shaded *P. thunbergii* seedlings through the enhancement of photosynthetic rates, water and Pi absorption, chlorophyll content, and carbon fixation, as well as through reductions of the LCP.

## Abbreviation

A: CO_2_ assimilation
AQY: Apparent quantum yield
CCP: CO_2_ compensation point
Chl: Chlorophyll
CSP: CO_2_ saturation point
ECM: Ectomycorrhizal
F_v_/F_m_: Maximal photochemical efficiency
LCP: Light compensation point
LSP: Light saturation point
NM: Non-mycorrhizal
Pi: Phosphorus
Pn: Photosynthetic carbon fixation
RWC: Relative water content
Φ_PS II_: Actual PS II efficiency
